# Mannose receptor 1 expression does not determine the uptake of high-density mannose dendrimers by activated macrophages populations

**DOI:** 10.1371/journal.pone.0240455

**Published:** 2020-10-13

**Authors:** Luciana Kovacs, Pablo Cabral, Roger Chammas

**Affiliations:** 1 Centro de Investigação Translacional em Oncologia, Instituto do Câncer do Estado de São Paulo, Faculdade de Medicina, Universidade de São Paulo, São Paulo, São Paulo, Brazil; 2 Departamento de Radiofarmacia, Centro de Investigaciones Nucleares, Facultad de Ciencias Universidad de la República, Montevideo, Uruguay; 3 Departamento de Radiologia e Oncologia da Faculdade de Medicina da Universidade de São Paulo, São Paulo, São Paulo, Brazil; Columbia University, UNITED STATES

## Abstract

The presence of a high number of macrophages within solid tumors is often significantly associated with poor prognosis and predict treatment failure for chemotherapy and radiotherapy. Macrophages are innate immune cells capable of performing diverse functions depending on the different signals from the microenvironment. The classically activated macrophage is commonly present during the early stages of tumor development while alternatively activated macrophages are associated with more advanced tumors. The distinction of the antitumoral macrophages from the pro-tumoral macrophages is not absolute. However, they have different cell surface markers such as mannose receptor (MRC1 or CD206) abundantly expressed by macrophages treated with interleukin-4 (IL-4). The important roles of macrophages in cancers suggest that it is important to develop novel therapies that target these cells. In the present study, we designed a probe using Polyamidoamine (PAMAM) fifth-generation (G5) dendrimers conjugated with mannose, Cyanine 7 (Cy7), and hydrazinonicotinamide (HYNIC) for target macrophages with high expression of MRC1 in the tumor. The intracellular uptake of ^99m^Tc-HYNIC-dendrimer-mannose-Cy7 through the interaction with MRC1 in bone marrow-derived macrophages (BMDMs) untreated or treated with lipopolysaccharides (LPS) + interferon (IFN)γ or IL-4 was analyzed. Our results show that high-density mannose dendrimers are preferentially bound by macrophages treated by IFNγ and LPS that express lower levels of MRC1 than for macrophages treated by IL-4 that express high levels of MRC1. Furthermore, the intracellular ^99m^Tc-HYNIC-dendrimer-mannose-Cy7 uptake in BMDMs was not inhibited in the presence of free mannose or glucose. This result suggests that ^99m^Tc-HYNIC-dendrimer-mannose-Cy7 is not internalized via macrophage MRC1. Based on these findings, we concluded that MRC1 expression does not determine the uptake of high-density mannose dendrimers.

## Introduction

Macrophages are components of the innate immune system. They play different roles in the maintenance of homeostasis, protection during infection, organ development, and contribute to some pathologic processes as chronic inflammatory diseases and cancer [1]. Macrophages are found in all tissues and display high functional diversity and plasticity [1–3]. They can change their functional state in response to changes in tissue physiology and microenvironmental signals [2]. Macrophages have different origins and roles. It was believed that the macrophage population in the adult was derived from the bone marrow and differentiated into tissue-specific macrophages in local tissues. However, some studies demonstrated that tissue-resident macrophages are derived from embryonic progenitors [3, 4]. During embryonic organogenesis, macrophages derived from the yolk sac and fetal liver precursors are seeded throughout tissues, persisting in the adulthood as resident macrophages. They are long-lived and self-maintain independently of the hematopoietic stem cells. After birth, bone marrow-derived monocytes can replenish tissue-resident macrophages following injury, inflammation, and infection [4].

In general, macrophages have been classified into two subsets, classically activated and alternatively activated exhibiting distinct phenotypes and functions. Classically activated macrophages induce an inflammatory and tumoricidal response. They are treated by LPS and IFNγ secreted by T helper 1 (Th1) lymphocytes or natural killer cells [5]. Alternatively activated macrophages are involved in the promotion of immunoregulatory functions, tissue remodeling, and tumor progression. They are treated by IL-4, secreted by T helper 2 (Th2) [5].

Tumor-associated macrophages (TAMs) represent a macrophage population recruited and educated by tumor cells and exist in almost all solid tumors [4]. They can account for up 50% of the tumor mass and have been implicated in tumor growth, angiogenesis, immunosuppression, invasion, and metastasis [4]. TAMs have been associated with poor prognosis and shorter survival in several types of cancer, including lung adenocarcinoma, ovarian and breast cancer [6–12]. TAMs are composed of distinct populations with features that depend on a variety of factors including the type of cancer, tumor stage, and location [10]. The heterogeneity of the TAM lineage has been recognized in the last decade. Although TAM was originally described as originating from circulating monocyte precursors released from the bone marrow; it was shown that some organs harbor embryonically derived populations of resident macrophages that self-maintain and self-renew throughout adulthood without the contribution of adult hematopoiesis [13–15]. Furthermore, the spleen constitutes an important extramedullary reservoir of monocytes that can supply growing tumors with macrophages. In most solid tumors, TAMs have functions and phenotypes similar to alternatively activated macrophages [5]. The distinction between classically and alternatively activated macrophages is not absolute. However, they present different cell surface markers, such as MRC1, a C-type lectin abundantly expressed by alternatively activated macrophages in mouse and human, which may be used as a target for molecular imaging probes [16, 17]. The MRC1 is a 180-kDa transmembrane glycoprotein with three types of extracellular domains. The extracellular region of the MRC1 contains an N-terminal cysteine-rich domain, a carbohydrate recognition domain, and a fibronectin II domain. It is an endocytic and phagocytic receptor that binds and internalizes glycoconjugates terminated in mannose, fucose, and collagen ligands [17]. Various are the roles of MRC1 such as clearance of endogenous molecules, cell adhesion, pathogen recognition, antigen presentation, modulation of cellular activation, and trafficking [17].

Several studies have investigated MRC1 as a potential target for imaging alternatively activated macrophages [18–23]. Sun et al. [22] prepared an anti-mouse CD206 antibody conjugated with DyLight680 succinimidyl ester, a fluorophore for near-infrared imaging, to target TAMs *in vivo*. The results showed an effective noninvasive visualization of TAMs in the 4T1 mouse breast cancer model. Movahedi et al. [18] developed a nanobody against MRC1 for target TAMs. ^99m^Tc-labeled anti-mannose receptor nanobodies could be successfully used to selectively target and image TAMs subpopulations *in vivo* using single-photon emission computed tomography (SPECT) imaging. Jiang et al. [19] described a compound prepared with D-mannosamine hydrochloride, a high-affinity ligand of the MRC1, conjugated with Cy7 to evaluate its ability to image TAMs in a hepatoma tumor. The results suggested that the compound was able to target TAMs and it can be a tool for tumor diagnosis. Li et al. [20] also demonstrated the effectiveness of an anti-biofouling PEG-b-AGE polymer-coated iron oxide nanoparticles for targeted imaging of MRC1 using a breast tumor model and magnetic resonance imaging. In addition, ^99m^TC-Tilmanocept is a radiopharmaceutical approved by the Food and Drug Administration for lymphatic mapping in solid tumors [21]. Tilmanocept is a synthetic nanomolecule, consisting of multiple units of mannose and diethylenetriaminepentaacetic acid (DTPA) attached to a dextran backbone, designed to target the MRC1 present on the surface of macrophages [21].

In the present study, our goal was to develop an imaging agent that selectively targets TAMs that express MRC1. For this purpose, we used PAMAM G5 dendrimers, a class of macromolecules synthetic, highly branched, and monodisperse with well-defined size and shape [24, 25]. They are formed of a multi-carbon ethylene diamine core and abundant numbers of the primary amino group on the surface, which allows doing a range of chemical modification to obtain specific biological properties. They are classified by generation number that is the number of focal points when going from the core towards the dendrimer surface [24]. PAMAM dendrimers have been widely used in biomedical applications in diagnosis and therapy [24–33]. Kulhari et al. [29] developed a PAMAM G4 dendrimer conjugated to trastuzumab, a recombinant monoclonal antibody, to improve docetaxel delivery to HER2 positive breast cancer cells. The results show that the compound improves the specific delivery of docetaxel and reduces systemic toxicity. Zhang et al. [30] also developed a PAMAM G5 dendrimer conjugated doxorubicin as a platform for pH-responsive drug release and specific targeting to cancer cells overexpressing folic acid receptors. The G5.NHAc-FA-DOX was able to specifically target cancer cells through folic acid receptors and showed significant therapeutic activity.

In this study, we described the designer of an imaging agent. PAMAM G5 dendrimers were conjugated with mannose, Cy7, and HYNIC, characterized by HPLC and MALDI-TOF, and the specific targeting of MRC1 in BMDM was examined.

## Materials and methods

Solvents, chemicals, and α-D-Mannopyranosylphenyl isothiocyanate (mannose) were purchased from Sigma-Aldrich (Milwaukee, WI, USA). PAMAM G5 dendrimers (ethylenediamine core, 128 amines (-NH_2_) end groups, 5 wt.% in methanol), and Lipopolysaccharides (LPS) were purchased from Sigma Aldrich (St Louis, MO, USA). Cyanine7 N-hydroxySuccinimide (Cy7-NHS) ester was purchased from Lumiprobe (Hunt Valley, MD, USA). Suc-HYNIC-tfa was synthesized at the Department of Organic Chemistry, University of the Republic, Montevideo, Uruguay, using the method previously described [34]. Sephadex G-25 in PD-10 desalting columns were purchased from GE Healthcare (Marlborough, MA, USA). Technetium-99m (^99m^Tc) was obtained from a ^99^Mo/^99m^Tc generator purchased from the Nuclear and Energy Research Institute (IPEN, Sao Paulo, Brazil). Roswell Park Memorial Institute (RPMI)-1640 medium and fetal bovine serum (FBS) were purchased from Gibco™ ThermoFisher Scientific (Waltham, MA, USA). Anti-mouse CD16/32 antibody (Cat #101302), PE anti-mouse F4/80 antibody (Cat #123110), and PE Rat IgG2a, k Isotype control (Cat #400508) were purchased from BioLegend (San Diego, CA, USA). IFNγ and IL-4 were purchased from R&D System (Minneapolis, MN, USA). TRIzol™ reagent, SYBR Green II RNA, High-Capacity cDNA Reverse Transcription Kits, SYBR Green Real-Time PCR Master Mixes, and StepOne System were purchased from Applied Biosystems, ThermoFisher Scientific (Waltham, MA, USA). Matrix-assisted laser desorption ionization-time of flight (MALDI-TOF) mass spectra were recorded on 4800 MALDI TOF/TOF (Abi Sciex) in the acquisition mode linear (linear Mid) as previously described by Bosnjakovic and coworkers [26].

### Synthesis of PAMAM G5 dendrimers-mannose-Cy7-HYNIC-Tfa

To perform conjugation, 8 mg of PAMAM G5 dendrimers solution in methanol was evaporated to remove methanol. PAMAM G5 dendrimers were dissolved in DMSO and 4.35 mg α-D-Mannopyranosylphenyl isothiocyanate was added and stirred for 1h at room temperature. PAMAM G5 dendrimers were labeled with α-D-Mannopyranosylphenyl isothiocyanate at an average molar ratio of 1:50. Subsequently, 3.31 mg of Cy7 NHS ester was added to the PAMAM G5 dendrimers-mannose and stirred for 1h at room temperature. PAMAM G5 dendrimers were labeled with Cy7 NHS ester at an average molar ratio of 1:20. After, 1.4 mg of Suc-HYNIC-Tfa was added to PAMAM G5 dendrimers-mannose-Cy7 and stirred for 1h at room temperature. PAMAM G5 dendrimers were labeled with Suc-HYNIC-Tfa at an average molar ratio of 1:10. After each successive addition reaction, the average degree of mannose, Cy7, and Suc-HYNIC-Tfa was quantified by MALDI-TOF. In this way, it was controlled the number of molecules per dendrimers. PAMAM G5 dendrimers-mannose-Cy7-HYNIC-Tfa were purified by gel filtration using a PD-1 desalting column containing Sephadex G25. Fractions of 0.5 mL were collected by using sodium acetate buffer 0,15 M (pH 8.3) as a mobile phase.

### Radiochemistry

For ^99m^Tc labeling, 9 mg of tricine was dissolved in 50 μL Hydrochloric acid (HCl) 0,1M and 800 μL of water. After mixing, it was added 50 μL of a fresh solution of stannous chloride dihydrate (10 mg dissolved in 500 μL HCl 0,1M) and 9 mL of 0,9% sodium chloride. 1 mg of—PAMAM G5 dendrimer-mannose-Cy7-HYNIC-Tfa was incubated with 50 μL of Tricine/stannous chloride dihydrate solution and 200 μL of Na^99m^TcO_4_ solution (250 MBq) for 1 hour at room temperature. The radiolabeled compound was purified by size exclusion chromatography (PD-10 column) and characterized by reverse-phase high-pressure liquid chromatography (RP-HPLC), Shimadzu, model SPD-10A VP, with gamma detector and UV/vis detector (λ = 214 nm) and Phenomenex Jupiter C18 column (250 x 4.60 mm, 4 μm). The mobile phase was 0,14% trifluoroacetic acid (TFA) in water (solvent A) and 0,14% TFA in acetonitrile (solvent B). The gradient method used for analysis was solvent A for the initial gradient and the proportion of the solvents was changed linearly reaching 0% solvent A and 100% solvent B over 20 min with a flow rate of 1 mL/min.

### *In vitro* stability

The radiolabeling stability of the ^99m^Tc-HYNIC-dendrimer-mannose-Cy7 *in vitro* was analyzed using PBS and histidine. ^99m^Tc-HYNIC-dendrimer-mannose-Cy7 (100 μL, 18.5 MBq) were incubated with 100 μL PBS or 100 μL 1mM of L-histidine in PBS solution for up to 3 hours at 37°C and analyzed by HPLC after 1, 2, and 3 hours.

### Macrophage cultures and *in vitro* experiments

All procedures were in accordance with ethical principles adopted by the Brazilian College of Animal Experimentation and approved by the Ethical Committee for Animal Research of School of Medicine, University of São Paulo (#014/15, 02/25/2015). Bone marrow was collected from eight-week-old male C57BL/6 mice. The mice were anesthetized with ketamine (150 mg/kg) and xylazine (10 mg/kg) administered intraperitoneally and sacrificed by cervical dislocation. Total cells were extracted from the femoral bone marrow. The bones were flushed with cold PBS until the bone cavity appears white. The bone marrow cells were cultivated in RPMI 1640 medium supplemented with 15% FBS and 30% of L-929 cells conditioned medium as a source of Macrophage Colony Stimulating Factor [35] for seven days at 37°C in a humidified incubator containing 5% CO_2_. On the seventh day, adherent cells were detached and analyzed for the purity of the cell population. After pre-treatment with Fc Block, 3 x 10^5^ cells were incubated with PE anti-mouse F4/80 antibody and PE Rat IgG2a, k isotype for 45 minutes at 4°C. The fluorescent signal was acquired with Attunes Cytometer (ThermoFisher Scientific, Waltham, MA, USA).

BMDMs were treated with 100 ng/mL LPS + 50 ng/mL IFNγ or 50 ng/mL IL-4 for 24 hours at 37°C in a humidified incubator containing 5% CO_2_. BMDMs activation was confirmed by evaluating the expression of nitric oxide synthase (iNOS), MRC1, and arginase 1 (Arg1) by quantitative reverse transcription-polymerase chain reaction (RT-PCR) analysis. Total RNA was isolated by TRIzol according to the manufacturer’s protocol. The integrity of the extracted RNA was verified via agarose gel electrophoresis. Intact, high-quality RNA was indicated by the presence of the two, bright 18S and 28S rRNA bands in SYBR Green II RNA stained agarose gels visualized under UV light. For each sample, approximately 1 μg of total RNA was converted to cDNA using High-Capacity cDNA Reverse Transcription Kits according to the manufacturer`s instructions. Quantitative RT-PCR was performed in 96 well plate using SYBR Green qPCR Master Mix following the manufacturer’s protocol, on the StepOne RT-PCR System. The primer sets used in this study were as follows: iNOS 5´-CAGGAACCTACCAGCTCACTCT-3´ (forward), 5´-ATGTGCTGAAACATTTCCTGTG-3´ (reverse); MRC1 5´-TTTGCAAGCTTGTAGGAAGGA-3´ (forward), 5´-CCAATCCACAGCTCATCATTT-3´ (reverse); Arg1 5´-GAACCCAACTCTTGGGAAGAC-3´ (forward), 5´-GGAGAAGGCGTTTGCTTAGTT-3´ (reverse); β-actin 5´-ACTGTCGAGTCGCGTCCA-3´ (forward), 5´-GCAGCGATATCGTCATCCAT-3´ (reverse). Data were analyzed using the StepOne Software (Version 2.1). The crossing point and cycle threshold were calculated by the second Derivate Maximum Method. The expression level of each mRNA was examined and normalized to the β-actin gene mRNA. The relative expression ratio of a target gene was calculated using the comparative CT method (2^-ΔΔCT^).

### Internalization assay

*In vitro* internalization assays were performed using BMDMs untreated and treated with LPS + IFNγ or IL-4. 100,000 cells were seeded onto a 24 well plate and incubated overnight. Briefly, approximately 0.037 MBq of ^99m^Tc-HYNIC-dendrimer-mannose-Cy7 was added to the cells and allowed to incubate for 1–6 hours at 37°C. After incubation, cells were washed with cold PBS, harvested and their radioactivity was measured using a 2480 Wizard automatic gamma counter from PerkinElmer (Waltham, MA, USA).

For the blocking study, BMDMs untreated and treated with LPS + IFNγ or IL-4 were incubated with a medium containing different concentrations of D-mannose or D-glucose for 30 minutes at 37°C. After the medium was replaced with 0.037 MBq of ^99m^Tc-HYNIC-dendrimer-mannose-Cy7 in medium, cells were incubated for 1h at 37°C. After the incubation, cells were washed with cold PBS, harvested and their radioactivity was measured as described above.

### Statistical analysis

Data were analyzed by unpaired t-test, one-way analyses of variance (ANOVA), or two-way ANOVA followed by posttest (Tukey-Kramer or Bonferroni). All analyses were conducted using GraphPad Prism version 4.0 for Windows^®^ (GraphPad^®^ software, San Diego, CA).

## Results

### Synthesis and characterization of PAMAM G5 dendrimers-mannose-Cy7-HYNIC-Tfa

**Fig 1:**
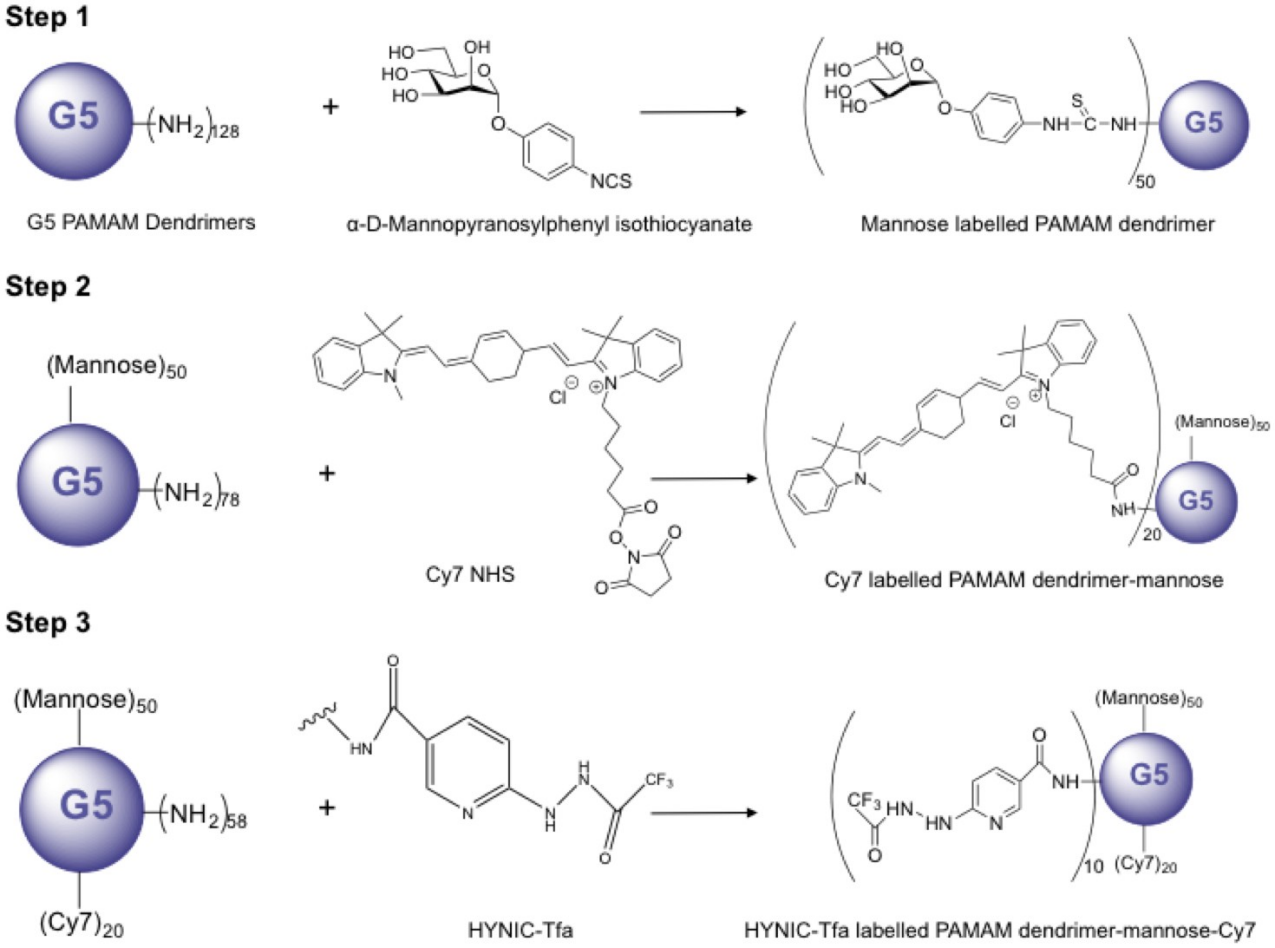
Schematic structure and synthesis method. In step 1, α-D-Mannopyranosylphenyl isothiocyanate was conjugated to -NH_2_ group of the dendrimer, using a ratio of 50:1 mannose to dendrimer. In step 2, Cy7 NHS ester was conjugated to -NH_2_ group of the dendrimer, using a ratio of 20:1 Cy7 to dendrimer. In step 3, Suc-HYNIC-Tfa was conjugated to -NH_2_ group of the dendrimer, using a ratio of 10:1 Suc-HYNIC-Tfa to dendrimer.

The goal of this study was to prepare and characterize a multifunctional PAMAM G5 dendrimer for identity alternatively activated macrophages within the tumor. The design of this compound is based on the PAMAM G5 dendrimers, which were used as the core structure of the compound. PAMAM G5 dendrimer-mannose-Cy7-HYNIC-Tfa was prepared through three steps (Fig 1).

**Fig 2:**
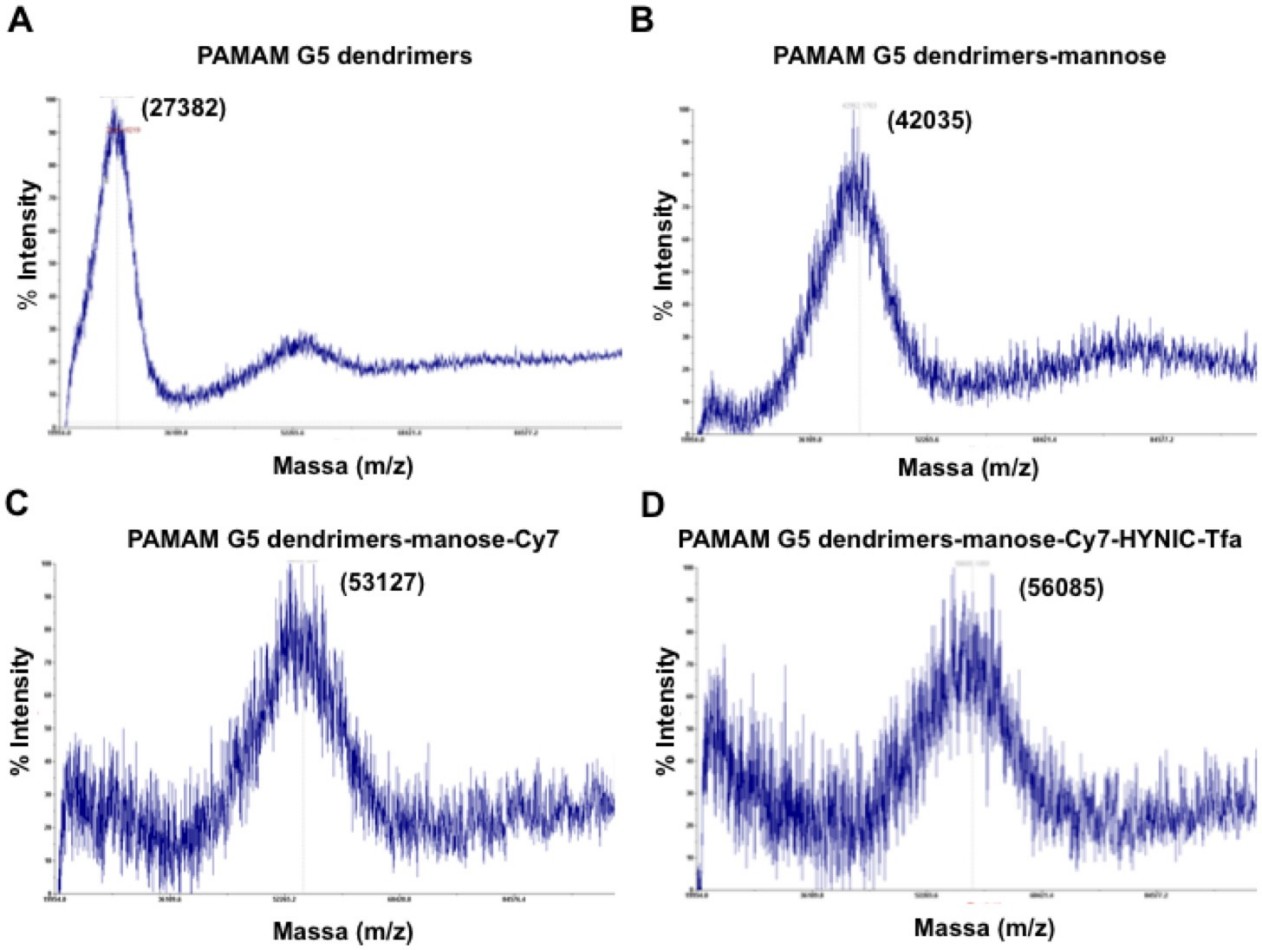
MALDI-TOF MS spectra. (A) PAMAM G5 dendrimers, (B) PAMAM G5 dendrimers-mannose, (C) PAMAM G5 dendrimers-manose-Cy7 and (D) PAMAM G5 dendrimers-manose-Cy7-HYNIC-Tfa.

In step 1, α-D-Mannopyranosylphenyl isothiocyanate was conjugated to the PAMAM G5 dendrimers by the reaction of the isothiocyanate group with -NH_2_ group of the dendrimer, this reaction created a stable thiourea bond. In step 2, Cy7 NHS ester was conjugated to the PAMAM G5 dendrimers-mannose. NHS ester crosslinked reacted with -NH_2_ group of the dendrimer and yielded stable amide bonds. Finally, in step 3 to chelate the nuclear probe ^99m^Tc, Suc-HYNIC-Tfa was coupled to the -NH_2_ group of the dendrimer via amide linkage [18]. The compound was characterized by MALDI-TOF mass spectrometry and general molecular weight distribution could be observed. The results suggest that the average number of terminal groups introduced in each step was approximate, 47 mannose, 16 Cy7, and 8 HYNIC-Tfa conjugated per dendrimer (Fig 2).

### Radiolabeling

**Fig 3:**
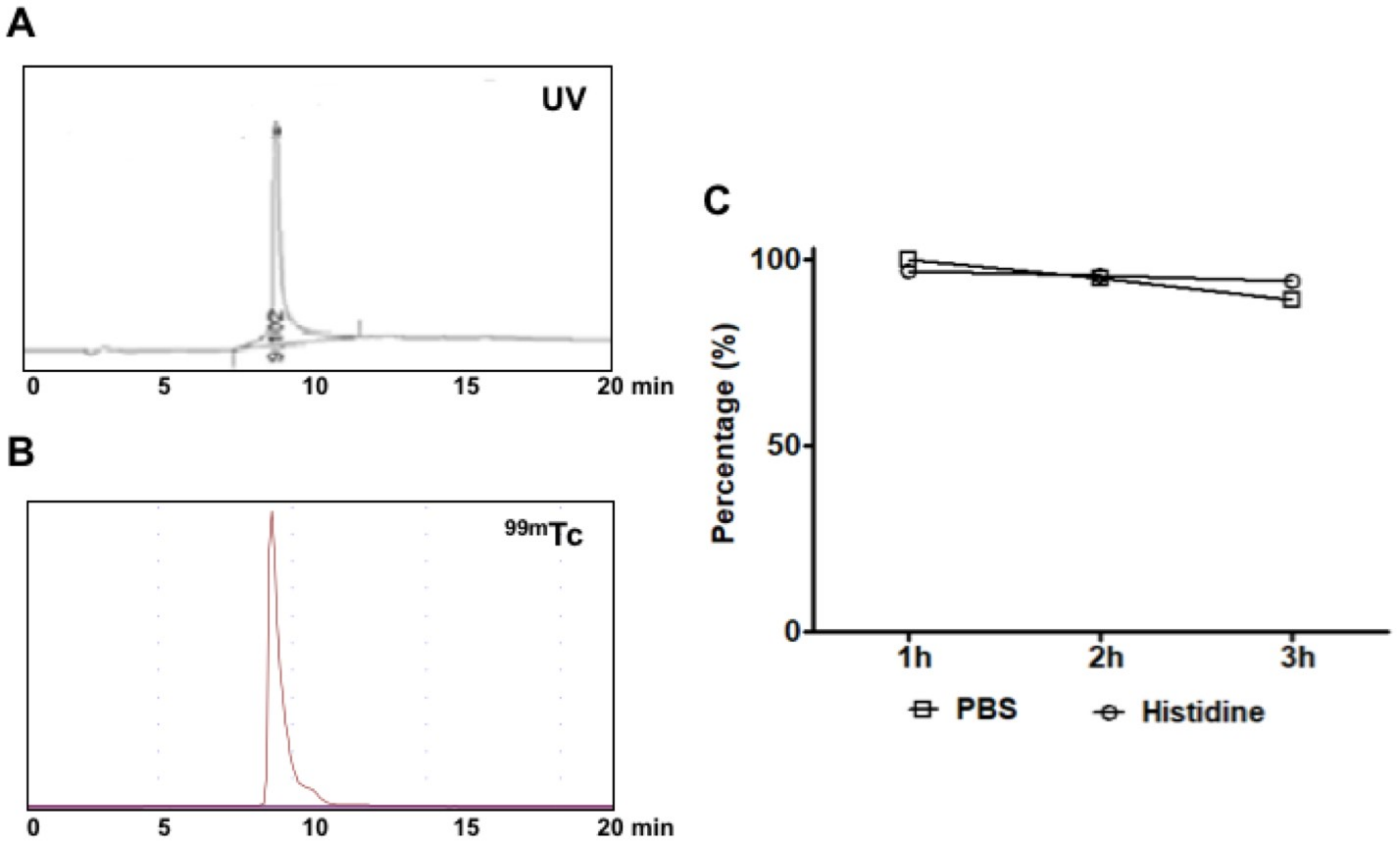
RP-HPLC analysis and stability. (A) RP-HPLC analysis of PAMAM G5 dendrimer-mannose-Cy7-HYNIC-Tfa UV (λ = 214) detector, the retention time was 9.10 min. (B) RP-HPLC analysis of radiolabeled ^99m^Tc-HYNIC-dendrimers-mannose-Cy7, the retention time was 9.20 min. (C) Stability of ^99m^Tc-HYNIC-dendrimers-mannose-Cy7 versus histidine and PBS. 100% was the total activity of the compound determined at time 0, n = 3 for each point on the graph.

^99m^Tc labeling was completed with the reaction of PAMAM G5 dendrimer-mannose-Cy7 HYNIC-Tfa and ^99m^TcO_4_^-^ (250 MBq) for 1 hour at room temperature. The radiolabeled compound was characterized by RP-HPLC. The ^99m^Tc-HYNIC-dendrimer-mannose-Cy7 showed excellent radiochemical yield (98%). The retention time of PAMAM G5 dendrimer-mannose-Cy7-HYNIC-Tfa was 9.10 minutes (Fig 3A) and of ^99m^Tc-HYNIC-dendrimer-mannose-Cy7 was 9.20 minutes, Fig 3B.

To ensure that the technetium label in ^99m^Tc-HYNIC-dendrimer-mannose-Cy7 was stable, the conjugated was incubated with 1 mM L-histidine in PBS solution for up to 3 hours at 37°C. The results show high stability, more than 94% in histidine, and 90% in PBS. The storage of ^99m^Tc-HYNIC-dendrimer-mannose-Cy7 in PBS or histidine for up to 3 hours did not cause technetium release from the conjugate, Fig 3C.

### BMDMs differentiation

**Fig 4:**
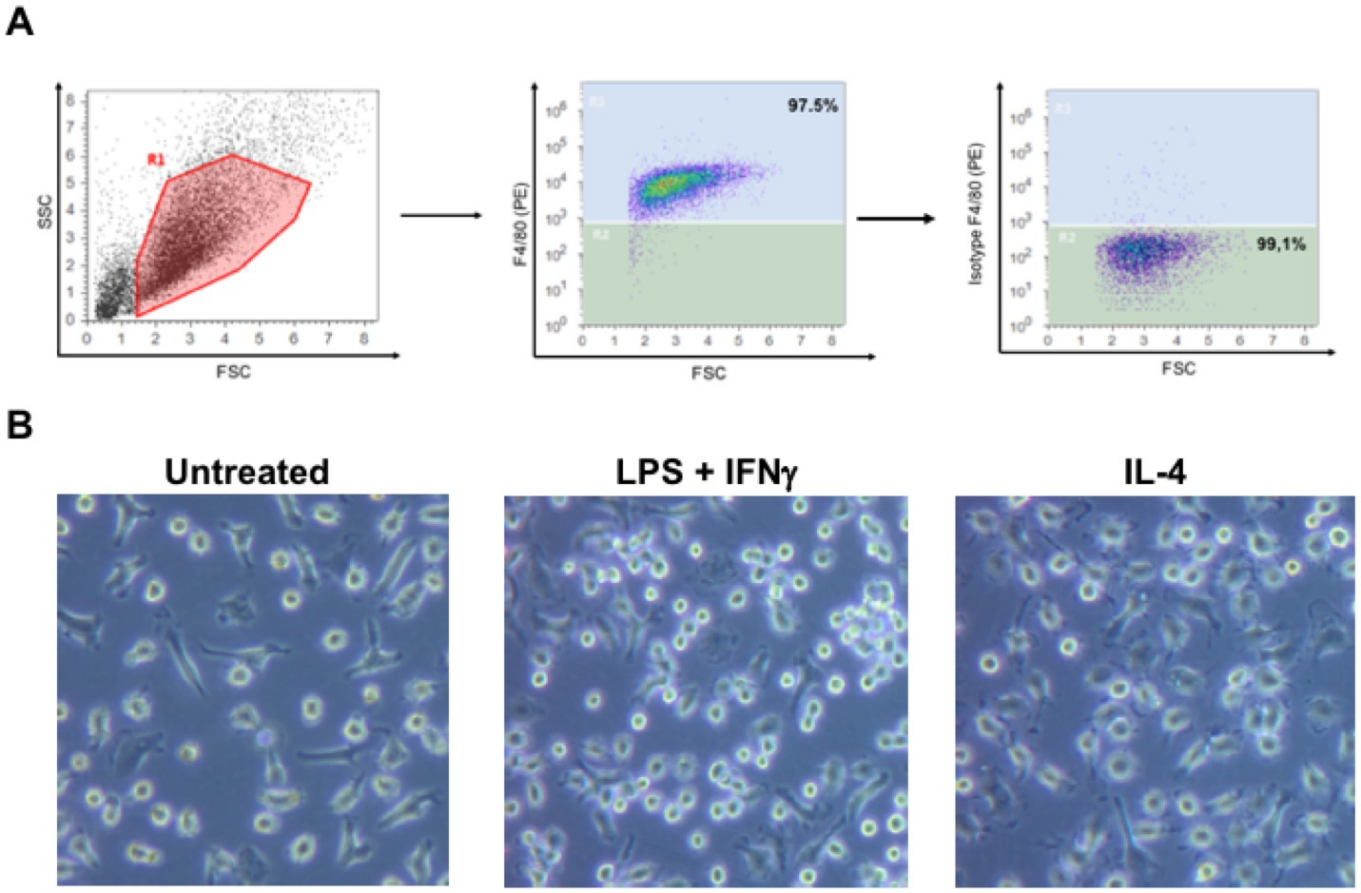
Flow cytometry analysis of BMDMs and differentiation. (A) BMDMs were first gated on FSC and SSC to remove debris and conjugates. Mature BMDMs were defined as F4/80+ subpopulations with the purity displayed as a percentage. (B) After treatment with LPS + IFNγ or IL-4 for 24 hours, BMDMs show different morphology.

Bone marrow cells were harvested from C57BL/6 mice and cultured in a medium containing macrophage colony-stimulating factor (M-CSF). After 7 days in culture, adherent cells were detached and flow cytometry analysis was performed. Mature BMDMs were defined as F4/80+ subpopulations. The binding frequency of the F4/80 antibody was 97.5%, Fig 4A. BMDMs were stimulated with 100 ng/mL LPS + 50 ng/mL IFNγ or 50 ng/mL IL-4 for 24 hours. BMDMs were observed under an optical microscope to evaluate the morphological changes, Fig 4B. The BMDMs treated with LPS and IFNγ, showed a rounder or dendritic morphology, and the BMDMs treated with IL-4, showed a rounder or spindle-shaped morphology.

**Fig 5:**
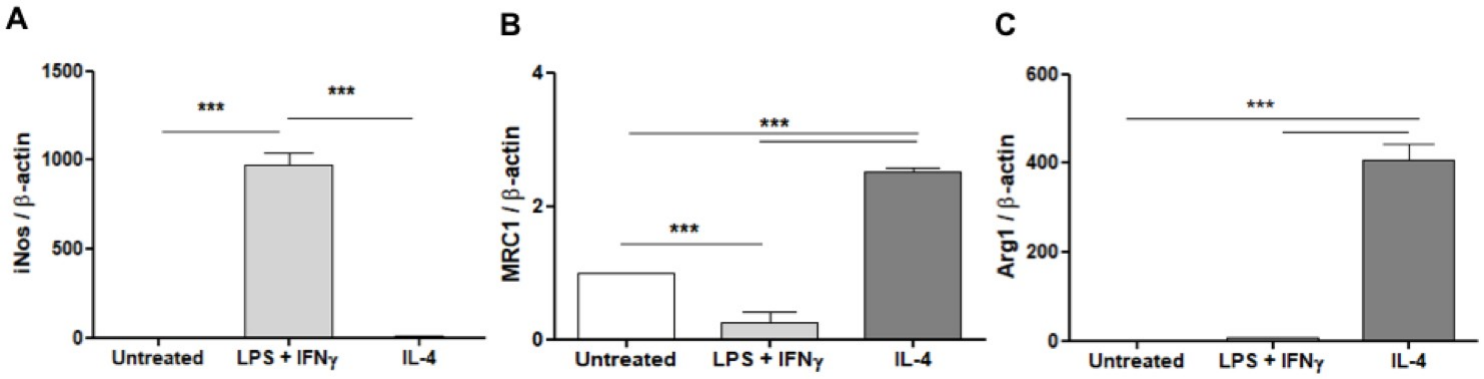
BMDMs polarization analysis. BMDMs were treated with LPS and IFN-γ or IL-4 for 24 hours. The polarization of BMDMs was confirmed by quantitative RT-PCR. (A) iNOS, (B) MRC1, and (C) Arg1 expression levels were normalized by β-actin (mean ± SD). A comparison in each group was performed by one-way ANOVA followed by Tukey-Kramer test (***p<0.0001).

The polarization of BMDMs with LPS + IFNγ or IL-4 was confirmed by quantitative RT-PCR. The iNos expression level was significantly higher in the BMDMs treated with LPS + IFNγ, Fig 5A. On the other hand, BMDMs treated with IL-4 showed significantly higher MRC1 and arginase 1 (Arg1) expression levels, Fig 5B and 5C.

### Cell uptake of ^99m^Tc-HYNIC-dendrimer-mannose-Cy7

**Fig 6:**
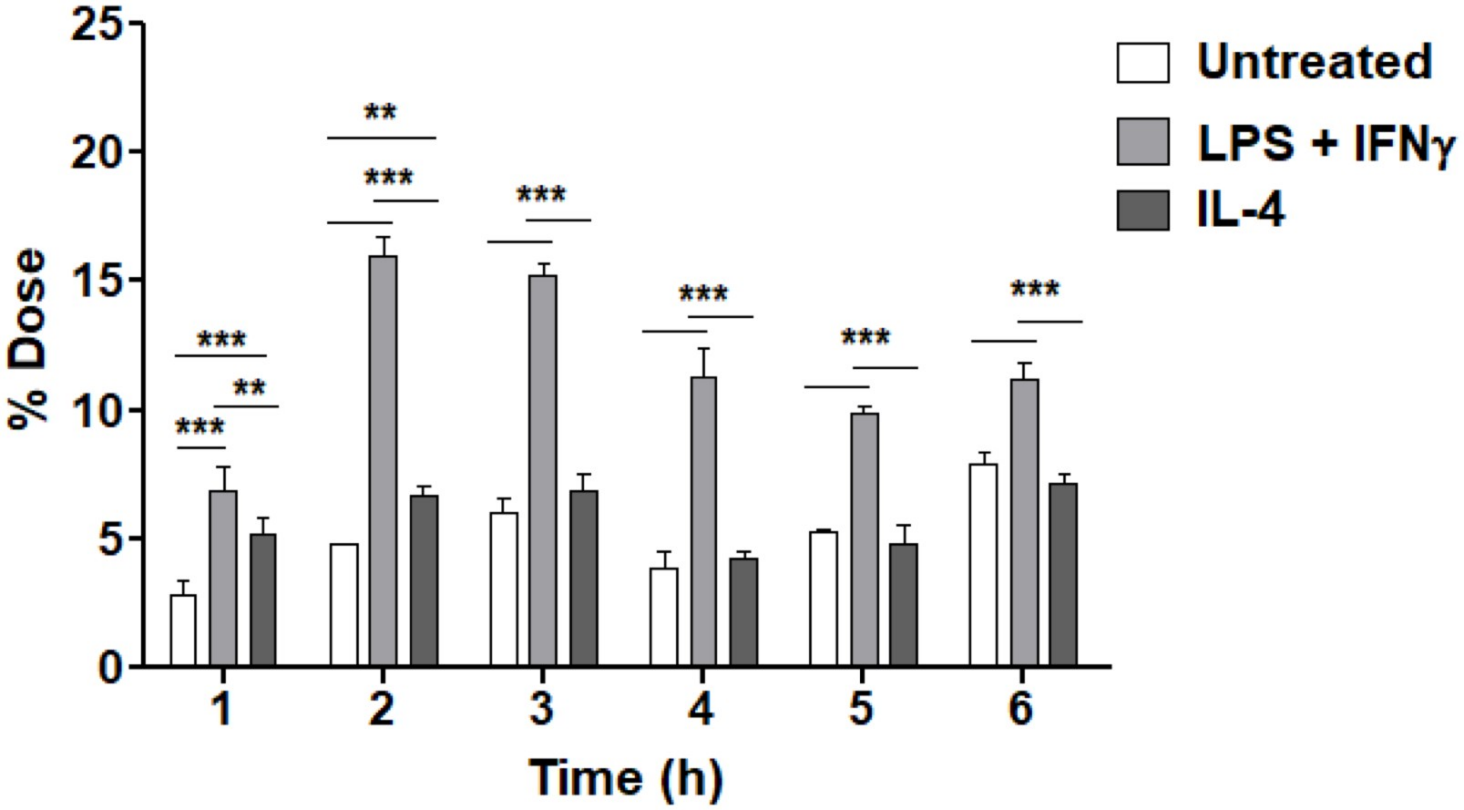
Cell uptake of ^99m^Tc-HYNIC-dendrimer-mannose-Cy7. BMDMs untreated and treated with LPS + IFNγ and IL-4 were incubated with ^99m^Tc-HYNIC-dendrimer-mannose-Cy7. Cellular uptake was analyzed 1 to 6 hours after incubation. Data are expressed as % dose (mean ± SD). A comparison in each group was performed by two-way ANOVA followed by the Bonferroni test (***p<0.001, **p<0.01 and ns = p>0.05).

*In vitro* internalization assays of ^99m^Tc-HYNIC-dendrimer-mannose-Cy7 were performed using BMDMs untreated and treated with LPS + IFNγ or IL-4. BMDMs treated with LPS + IFNγ showed significantly higher levels of ^99m^Tc-HYNIC-dendrimer-mannose-Cy7 than BMDMs untreated or treated with IL- 4, Fig 6. The higher uptake of ^99m^Tc-HYNIC-dendrimer-mannose-Cy7 in BMDMs treated with IL-4 was observed after incubating cells for 6 hours, [7.22 ± 0.32] vs [10.86 ± 0.48] (BMDMs treated with LPS + IFNγ), [7.70 ± 0.36] (BMDMs untreated) % dose.

**Fig 7:**
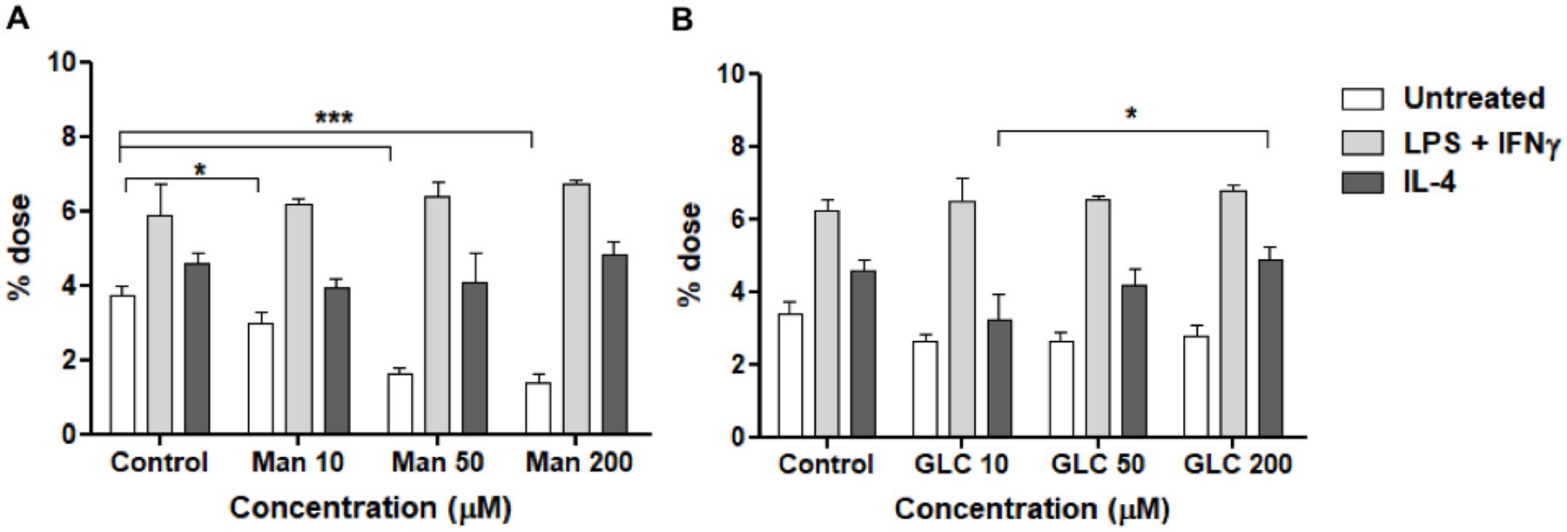
Cell uptake of ^99m^Tc-HYNIC-dendrimer-mannose-Cy7 with block. (A) BMDMs uptake of ^99m^Tc-HYNIC-dendrimer-mannose-Cy7, cells treated with different concentrations of D-mannose. (B) BMDMs uptake of ^99m^Tc-HYNIC-dendrimer-mannose-Cy7, cells treated with different concentrations of D-glucose. Data are expressed as % dose (mean ± SD). A comparison in each group was performed by two-way ANOVA followed by the Bonferroni test (*p < 0.005). Ctr (control), Man (mannose), Glc (glucose).

To evaluate whether ^99m^Tc-HYNIC-dendrimer-mannose-Cy7 uptake in BMDMs depends on the MRC1, the cellular uptake of ^99m^Tc-HYNIC-dendrimer-mannose-Cy7 was studied with or without block with α-D-mannose. The ^99m^Tc-HYNIC-dendrimer-mannose-Cy7 accumulation in BMDMs treated with IL-4 were not significantly suppressed by mannose blocking [4.59 ± 0.30 (no blocking) vs 3.91 ± 0.27 (10 μM mannose blocking), 4.07 ± 0.81 (50 μM mannose blocking) and 4.85 ± 0.37 (200 μM mannose blocking), Fig 7A. In BMDMs treated with LPS + IFNγ, ^99m^Tc-HYNIC-dendrimer-mannose-Cy7 accumulation increased with mannose blocking [5.91 ± 0.85 (no blocking) vs 6.20 ± 0.13 (10 μM mannose blocking), 6.41 ± 0.36 (50 μM mannose blocking) and 6.70 ± 0.10 (200 μM mannose blocking), Fig 7A. ^99m^Tc-HYNIC-dendrimer-mannose-Cy7 accumulation in BMDMs untreated was significantly suppressed by mannose blocking [3.73 ± 0.25 (no blocking) vs 2.98 ± 0.28 (10 μM mannose blocking), 1.61 ± 0.14 (50 μM mannose blocking) and 1.38 ± 0.27 (200 μM mannose blocking), Fig 7A.

To evaluate whether macrophage MRC1 was blocked specifically by mannose, the cellular uptake of ^99m^Tc-HYNIC-dendrimer-mannose-Cy7 was studied with or without D-glucose block. The accumulation of ^99m^Tc-HYNIC-dendrimer-mannose-Cy7 in BMDMs treated with IL-4 was not suppressed by glucose blocking 4.59 ± 0.30 (no blocking) vs 3.27 ± 0.71 (10 μM glucose blocking), 4.20 ± 0.45 (50 μM glucose blocking) and 4.87 ± 0.35 (200 μM glucose blocking), Fig 7B. Likewise, the accumulation of ^99m^Tc-HYNIC-dendrimer-mannose-Cy7 was also not suppressed by glucose blocking in BMDMs stimulated with LPS + IFNγ 5.90 ± 0.85 (no blocking) vs 6.50 ± 0.68 (10 μM glucose blocking), 6.50 ± 0.10 (50 μM glucose blocking) and 6.80 ± 0.16 (200 μM glucose blocking) and BMDMs untreated 3.25 ± 0.35 (no blocking) vs 2.62 ± 0.20 (10 μM glucose blocking), 2.62 ± 0.24 (50 μM glucose blocking) and 2.78 ± 0.28 (200 μM glucose blocking), Fig 7B.

## Discussion

Solid tumors are composed of several types of cells such as fibroblasts, endothelial, and a variety of immune cells that create a unique microenvironment. TAMs are defined as macrophages residing within the tumor. They interact with tumor cells and elements from the tumor stroma. The presence of macrophages in solid tumors is correlated with poor prognosis in different cancers. They promote metastasis through several mechanisms, including the promotion of angiogenesis, induction of tumor growth, enhancement of tumor cell migration, and invasion by releasing chemokines, cytokines, and proteases that modify the tumor microenvironment. The important roles of macrophages in cancers suggest them as potential targets for new diagnostic procedures and new therapies. However, the targeted imaging of TAMs is still limited by the lack of imaging agents, which could also be applied as vectors for specific delivery of therapeutic drugs.

The primary objective of this work was to develop a multimodal probe that simultaneously offers complementary forms of hybrid image, specifically, optical image and SPECT to identify TAMs into the tumor. The optical images provide sensitive detection of superficial lesions while SPECT images provide quantitative and sensitive detection of deeper structures. In this study, we evaluated the utility of MRC1 as a target for imaging macrophages stimulated with IL-4 *in vitro*. To develop a multimodal probe, we purchased PAMAM G5 dendrimers and modified their surface with an α-D-Mannopyranosylphenyl isothiocyanate. The free amino group of dendrimers reacted with the functional group isothiocyanate and made a new functional group Thiourea stable. We chose PAMAM G5 dendrimers because they have a well-defined multifunctional surface (128 primary amines) and many different compounds such as mannose, fluorophores, and radionuclides may be conjugated at their surfaces to obtain specific properties. Furthermore, other properties associated with PAMAM dendrimers such as monodispersity, nanoscale shape, and size, water-solubility, chemical stability, low cytotoxicity, high ligand density, and multifunctionality make them attractive to design a multimodal probe [32].

For the optical image, we chose the near-infrared Cy7 dye (Ex. 750 nm; Em.773 nm). Near-infrared optical imaging is a powerful tool for studying the molecular events in solid tumors and for diagnosing the biological processes *in vivo*. It can be used to image a variety of molecular features due to its versatile fluorescent probe design, it is highly sensitive, it can provide dynamic, real-time *in vivo* images and it is inexpensive [36]. Near-infrared light can penetrate several centimeters into the tissue, it is potentially safe and a valuable tool for studying the status of receptor expression noninvasively in small animals. For SPECT imaging, we chose ^99m^Tc because it has good radiation physical characteristics (Isometric transition (IT), 140 keV, half-life 6 hours) and easy availability at low cost through ^99^Mo/^99m^Tc generator [24, 33]. The radiolabeling of dendrimers with ^99m^Tc was indirectly achieved through Suc-HYNIC-Tfa an efficient bifunctional chelator often used to synthesize bioconjugates for radiolabeling with ^99m^Tc [24]. Because HYNIC occupies only one or two coordination positions of the octahedral coordination sphere of ^99m^Tc, co-ligand tricine was used to stabilize the molecule. The MALDI-TOF mass analyses suggested successful synthesis of HYNIC-dendrimer-mannose-Cy7, the conjugate ^99m^Tc-HYNIC-dendrimer-mannose-Cy7 showed excellent radiochemical yield 98%. An *in vitro* stability study of ^99m^Tc-HYNIC-dendrimer-mannose-Cy7 was performed by incubation and competition assay with histidine. PBS solution was used as control. Radiolabeled dendrimer was stable in histidine until 3 hours with 94% of the ^99m^Tc bound to the dendrimer.

The specific binding potential of ^99m^Tc-HYNIC-dendrimer-mannose-Cy7 to macrophages was analyzed. Macrophages perform different functions upon stimulation with different factors. In this study, the phenotype of BMDMs cells was characterized by evaluating the gene expression levels of the iNOS and Arg1. BMDM cells treated with LPS + IFNγ showed significantly increased iNOS expression levels while those treated with IL-4 showed significantly increased Arg1 expression levels when compared with untreated cells. We also investigate the effect on the MRC1 expression levels after stimulated with LPS + IFNγ or IL-4. The MRC1 expression level was upregulated in macrophages treated with IL-4 and significantly downregulated in macrophages treated with LPS + IFNγ, compared with the untreated macrophages.

The intracellular uptake of ^99m^Tc-HYNIC-dendrimer-mannose-Cy7 was evaluated by radioactive internalization assay. We observed that, compared to untreated cells, the intracellular uptake was increased significantly in macrophages treated with LPS + IFNγ. The intracellular uptake of macrophages treated with IL-4 was significantly higher compared to untreated cells at 1 and 2 hours of incubation, but no significant difference was observed at 3 to 6 hours. However, the uptake of macrophages treated with LPS + IFNγ, IL-4 and untreated cells decreased at 4 hours, and uptake increased again at 6 hours. The highest uptake of ^99m^Tc-HYNIC-dendrimer-mannose-Cy7 in macrophages treated with IL-4 and untreated was observed after incubation of the cells for 6 hours. These results suggest that the compound may enter an exocytosis route or induce cell toxicity. The endocytosis and exocytosis are simultaneous and dynamic processes dependent on each other [37]. Several studies have analyzed cell internalization and trafficking of dendrimers [32, 38–41] but little is known about exocytosis of PAMAM dendrimers from cells. Sakhtianchi et al. [37] analyzed the exocytosis of different nanoparticles and described several factors that may affect the exocytosis process, such as size, shape, surface properties, nanoparticle concentration, incubation time, and cell type. In addition, PAMAM dendrimers have been described in a wide range of biomedical applications, and their cytotoxicity has been analyzed using different mammalian cell lines. Mukherjee et al. [42] study the cytotoxicity of unmodified PAMAM dendrimers in human keratinocytes through the evolution of reactive oxygen species (ROS), caspase activation, and inflammation response among other analyzes. The toxicity of PAMAM dendrimers depends on the dendrimer generation and dose [42]. Naha et al. [43] evaluated the immunotoxicity of PAMAM dendrimers of different generations (G4, G5, G6) *in vitro* using J774A.1, a macrophage cell line. The immunotoxicity response of PAMAM dendrimers depends on the size of the dendrimer (G6 > G5 > G4) and the number of primary amino groups present on the surface.

Blocking studies with mannose solution showed a significant decrease in intracellular uptake of ^99m^Tc-HYNIC-dendrimer-mannose-Cy7 in untreated macrophages and no differences in the intracellular uptake of macrophages treated with LPS + IFNγ or IL-4 were observed. We also performed blocking studies using a glucose solution, no significant difference in the intracellular uptake of macrophages treated with LPS + IFNγ or IL-4 or untreated macrophages were observed. These results suggest that dendrimers with high mannose density enter the cells not only by MRC1 but also through other pathways.

The internalization of nanoparticles into cells and intracellular transport also depends on their physicochemical properties, such as overall charge, size, composition, shape, surface modification properties, and the cell type [32]. In general, when the nanoparticles reach the cell, they are internalized by endocytosis. PAMAM dendrimers enter the cell by caveolae-dependent endocytosis and micropinocytosis pathways [40, 41]. However, PAMAM dendrimers can be modified by attaching target-specific ligands in their surfaces, these ligands recognize specific receptors over-expressed on the target cells. Modified dendrimers when reaching cells can be internalized through receptor-mediated endocytosis, only receptor-specific molecules can enter the cell through this process [32]. In this study, dendrimers were modified with mannose, as these mannoses recognize MRC1 on the surface of macrophages treated with IL-4, modified dendrimers rich in mannoses can enter the cells by MRC1. Our results suggest that the conjugation of mannose to the surface of the dendrimer gives them the ability to target MRC1, but not impeding the dendrimer to use other endocytic pathways to enter cells. In addition, macrophages are professional phagocytes and macrophages treated with LPS + IFNγ exhibit a high level of phagocytic activity, this can explain the high level of internalization of ^99m^Tc-HYNIC-dendrimer-mannose-Cy7 in these cells. The strategy of using dendrimers rich in mannose was not useful for specific target of macrophages treated with IL-4 in the tumor microenvironment.

Although several studies have shown the use of different agents binding successfully to the MRC1 in TAMs, little is known about the direct binding and internalization pathways of these compounds through MRC1 [18, 19, 22, 23]. Sharma et al. [44] developed a PAMAM G4 dendrimer conjugated with mannose and cyanine 5 (Cy5) and investigated the ability of the RAW 264.7, a murine macrophage cell line, treated with LPS or IL-4 to internalize the Mannose-dendrimer-Cy5 after 8 hours incubation. The results suggested that the conjugation of mannose to the PAMAM G4 dendrimers surfaces changes the mechanism of dendrimer internalization, and enhanced their ability to target MRC1 mediated endocytosis. However, the results showed uptake of Mannose-dendrimer-Cy5 for RAW 264.7 treated with LPS and IL-4 and indicate that the compound was not specific uptake for MRC1. He et al. [45] also developed PAMAM G5 dendrimer conjugated with mannose and LXR-LT091317. The objective was to develop an agent for specific delivery to atherosclerotic plaque-associated macrophages via MRC1. Cellular uptake was assessed by fluorescent imaging using a mouse peritoneal macrophages and primary mouse hepatocytes. The results indicated an increase in the uptake of dendrimers conjugated with mannose by macrophages, demonstrating specific macrophages targeting. These studies confirm our results, PAMAM dendrimers conjugated with mannose can bind macrophages. However, the compound is not exclusively uptake by MRC1 and the level of uptake cannot differentiate macrophages that express low levels of MRC1 from macrophages that express high levels of MRC1 which was the main objective of this project.

## Conclusion

Here, we could show that dendrimers can indeed be used as carrier molecules for imaging agents with good radiochemical yield and stability. Our hypothesis that we could exploit a functional feature of macrophages treated with IL-4 related to the overexpression of MRC1, however, proved false. The high density of mannose residues on dendrimers seems to be potentially recognized not only by higher affinity MRC1 but a variety of other endocytic molecules that recognize the array of carbohydrates that modify the surface of PAMAM G5 dendrimers. Our study does allow for a cautionary note that emphasizes the need to consider internalization mechanisms of complex molecules that may be used as imaging probes for professional phagocytes, such as macrophages.
